# Impact of Anesthesia Modality on Clinical Outcomes in Anterior Circulation Stroke Patients Undergoing Mechanical Thrombectomy: A Retrospective Propensity Score-Matched Analysis

**DOI:** 10.3390/jcm15134916

**Published:** 2026-06-24

**Authors:** Mustafa Çetiner, Ayşe Nur Akca, Gönül Akdağ, Buse Kozlu, İrem Yıldırım, Abdulhamit Şehid Kılınç, Esma Aşık, Burcu Kaplan, Fahri Şen, Nurullah Can Aydoğdu

**Affiliations:** 1 Department of Neurology, Faculty of Medicine, Kütahya Health Sciences University, Kütahya 43000, Türkiye; akcaaysenur95@gmail.com (A.N.A.); gonul.akdag@ksbu.edu.tr (G.A.); irem.yildirim@ksbu.edu.tr (İ.Y.); nurullahcan4242@gmail.com (N.C.A.); 2 Department of Anesthesiology and Reanimation, Kütahya Evliya Çelebi Training and Research Hospital, Kütahya 43000, Türkiye; busedeveli@gmail.com (B.K.); drabdulhamitkilinc@gmail.com (A.Ş.K.); drburcukaplann@gmail.com (B.K.); drfahri@gmail.com (F.Ş.); 3 Department of Algology, Faculty of Medicine, Ege University, İzmir 35000, Türkiye; esmaasik91@outlook.com

**Keywords:** ischemic stroke, anesthesia, thrombectomy

## Abstract

**Background/Objectives:** The type of anesthesia in patients with acute stroke is still controversial. This study aimed to investigate the effect of anesthesia management on clinical outcomes in patients with anterior circulation stroke undergoing mechanical thrombectomy. **Methods:** In this observational, retrospective study, patients with acute anterior circulation stroke who underwent mechanical thrombectomy between January 2021 and March 2025 were retrospectively reviewed. Patients were divided into groups according to the type of anesthesia administered. Functional independence was assessed at 90 days using the modified Rankin Scale. A TICI of 2b or higher was defined as successful reperfusion. In the conscious sedation (CS) and general anesthesia (GA) groups, patients were analyzed based on baseline characteristics and clinical outcomes using Propensity Score Matching. **Results:** In the propensity score-matched cohort, there was no significant difference between the two groups in terms of good functional outcome (mRS ≤ 2) (*p* = 0.82). When compared by successful reperfusion rate (TICI 2b or higher), it was significantly higher in the GA group (*p* = 0.01). **Conclusions:** In this study, the use of GA resulted in higher recanalization rates in patients with anterior circulation stroke undergoing mechanical thrombectomy. However, no differences were observed between the GA and CS groups in terms of functional outcomes, mortality, or peri-procedural/post-procedural complications.

## 1. Introduction

Ischemic stroke is the most common type of stroke worldwide and accounts for approximately 68% of all strokes [1,2]. Patients admitted to neurology clinics with acute ischemic stroke may receive intravenous thrombolytic therapy, endovascular treatment, or combined therapy during the acute period. Recent studies have demonstrated that endovascular treatment is superior to thrombolytic therapy in patients with major vessel occlusion detected on imaging, and mechanical thrombectomy (MT) has become the gold standard of care in current clinical practice [3,4].

The purpose of treatment is to recanalize the occluded vessel and salvage the ischemic penumbra. Although many factors affect long-term mortality and morbidity, one of the most important determinants is symptom-to-recanalization time [2]. Therefore, anesthesia selection during mechanical thrombectomy (MT) is an important factor for minimizing this duration and maintaining hemodynamic stability. General anesthesia (GA), conscious sedation (CS), or local anesthesia may be preferred during MT. Although there is no clear consensus on anesthesia selection, the patient’s clinical condition and the experience of the anesthesiologist and neurologist are considered [5]. Both GA and CS have advantages and disadvantages. Airway protection, lower aspiration risk, and patient immobilization during the procedure are considered advantages of GA. Therefore, higher reperfusion rates have been reported in patients undergoing GA [6,7]. However, GA may also complicate neurological examination during the procedure, delay puncture and recanalization times because of premedication before intubation, and increase hospitalization due to intubation-related complications and prolonged extubation periods [8,9]. Conscious sedation has advantages such as shorter preparation time and early recognition of neurological changes during the procedure. However, patient movement during the procedure may prolong procedure duration and increase mechanical vascular complications [9,10]. Several studies comparing GA and CS have been conducted; however, no definite conclusion has been reached. Earlier studies generally reported worse neurological outcomes in patients treated under GA, whereas more recent studies demonstrated that GA was not inferior to CS [9]. In recently published meta-analyses, general anesthesia is associated with higher rates of successful reperfusion; however, there is no consistent difference between anesthesia methods in terms of functional outcomes and mortality [11,12]. The objective of our study was to investigate the effect of anesthesia modality on clinical outcomes in patients with acute ischemic stroke undergoing endovascular treatment.

## 2. Materials and Methods

### 2.1. Study Cohort and Data Collection

**Figure 1:**
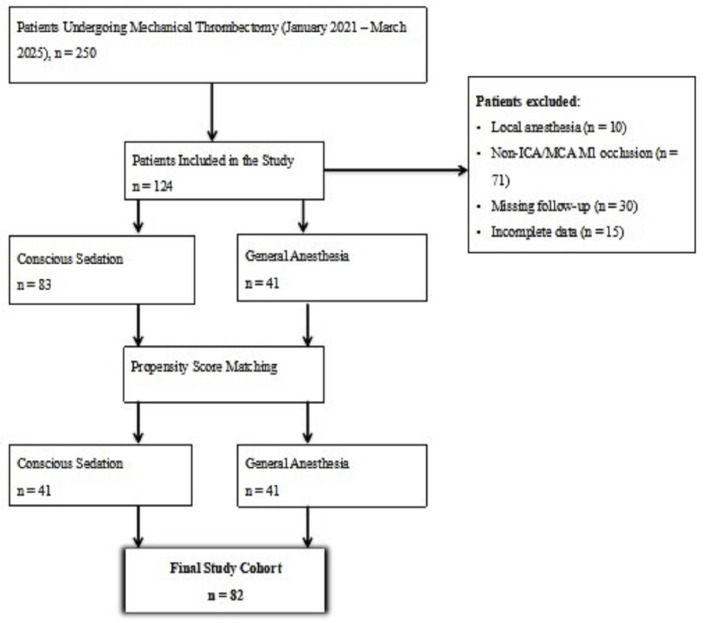
Flowchart of the study cohort.

In this observational, retrospective study conducted at the stroke center of Kütahya Evliya Çelebi Training and Research Hospital, patients with acute ischemic stroke who underwent MT between January 2021 and March 2025 were retrospectively reviewed. During the study period, there were no major changes in the mechanical thrombectomy devices (Artis Zee system, Siemens Healthineers, Erlangen, Germany) used at our center, in the interventional treatment approach, or in the fundamental principles of anesthesia management that could have influenced the study outcomes. A total of 250 patients who underwent MT were recorded. Accordingly, 10 patients treated under local anesthesia, 71 patients with occlusions other than the Internal Carotid Artery terminus or the Middle Cerebral Artery M1 segment, 30 patients for whom 3-month follow-up data were unavailable due to non-attendance at scheduled follow-up visits, and 15 patients with incomplete clinical data were excluded from the study. Consequently, 124 patients were included in the final analysis. The flowchart of the study cohort is presented in Figure 1. The GA and CS groups consisted of 41 and 83 patients, respectively. Clinical data were recorded by neurologists working in the stroke center. The choice of anesthesia type (GA or CS) and the anesthetic agents used were determined by the anesthesiologist. The selection of anesthesia was based on the neurological and general condition of the patient, including preprocedural intubation, impaired level of consciousness, agitation, aspiration risk, vomiting, and respiratory failure. Patients undergoing MT were divided into two groups: those who received GA with endotracheal intubation and those who received CS with local anesthesia. All patients in the general anaesthesia group received a standardized volatile-based anaesthetic regimen. Patients undergoing general anaesthesia received intravenous midazolam (0.02–0.05 mg·kg^−1^), lidocaine (1–1.5 mg·kg^−1^), propofol (2–3 mg·kg^−1^), fentanyl (1–2 μg·kg^−1^), and rocuronium (0.6 mg·kg^−1^) for induction. Anaesthesia was maintained with sevoflurane in an oxygen-air mixture. When deemed clinically necessary by the attending anaesthesiologist, a remifentanil infusion was administered intraoperatively to optimize haemodynamic stability and procedural conditions. Post-treatment symptomatic intracerebral hemorrhage (sICH) was defined according to ECASS III criteria [13]. Demographic characteristics, vascular risk factors, symptom-to-puncture time, puncture-to-recanalization time, and symptom-to-recanalization time were recorded. Patients were grouped according to the etiological classification of the Trial of Stroke Org 10,172 in Acute Stroke Treatment (TOAST) [14]. Pretreatment brain computed tomography (CT) ASPECT scores (cortical and subcortical), collateral scores on computed tomography angiography (CTA), and the number of passes during MT were recorded. Clinical outcomes, including National Institutes of Health Stroke Scale (NIHSS) scores at admission and discharge, in-hospital mortality, duration of hospitalization, and modified Rankin Scale (mRS) scores at 3 months after stroke, were evaluated. A modified Rankin Scale (mRS) score ≤2 at 3 months was accepted as a good functional outcome. According to the Thrombolysis in Cerebral Infarction (TICI) reperfusion category, TICI 2b, 2c, and 3 were defined as successful reperfusion [15]. Complications occurring during and after MT, including pneumonia, embolization to a different vessel, perforation, and dissection, as well as the number of patients undergoing decompressive surgery, were recorded. In patients without pneumonia at admission, pneumonia developing at least 48 h after hospitalization was classified as a complication. The diagnosis was established according to hospital-acquired pneumonia diagnostic criteria based on clinical, radiological, and laboratory findings, including elevated inflammatory markers such as C-reactive protein and procalcitonin [16]. Demographic data and clinical outcomes were compared between the GA and CS groups.

### 2.2. Statistical Analysis

Data were analyzed using R software version 4.4.1. Normality of the variables was evaluated using the Kolmogorov–Smirnov test. CS and GA groups were matched according to baseline characteristics and clinical outcomes using Propensity Score Matching (PSM). Propensity score matching was performed to reduce selection bias between the conscious sedation and general anesthesia groups. All baseline variables presented in Table 1 were included in the propensity score model as potential confounders. Propensity scores were estimated using logistic regression with anesthesia type as the dependent/grouping variable. Patients were matched at a 1:1 ratio without replacement, resulting in 41 matched pairs. Covariate balance before and after matching was assessed using absolute standardized mean differences. Balance diagnostics are provided in Supplementary Table S1. Associations between categorical variables were evaluated using Fisher’s Exact test, Yates correction, Monte Carlo corrected Fisher’s Exact test, and Pearson’s Chi-square test. Multiple comparisons were performed using a Bonferroni-corrected Z test. The Mann–Whitney U test was used for non-normally distributed variables, while the Independent Samples *t* test was used for normally distributed variables. Categorical variables were expressed as frequencies and percentages. Quantitative variables were expressed as mean ± standard deviation and median (minimum: maximum). A *p*-value < 0.05 was considered statistically significant.

## 3. Results

### 3.1. Demographic and Baseline Characteristics of Patients

Among 124 patients with acute ischemic stroke who underwent MT, 83 patients (67%) underwent MT under CS, and 41 patients (33%) underwent MT under GA. Median age was 69 years in the CS group and 70 years in the GA group. There was no significant difference between the groups in terms of age (*p* = 0.49). In the CS group, 38 patients (45.8%) were male, and 45 patients (54.2%) were female. In the GA group, 20 patients (48.8%) were male, and 21 patients (51.2%) were female. There was no statistically significant association between sex and anesthesia modality (*p* = 0.90). After PSM median age remained 69 years in the CS group and 70 years in the GA group. There was no significant difference between the groups (*p* = 0.97). In the matched cohort, 19 patients (46.3%) in the CS group and 20 patients (48.8%) in the GA group were male. No statistically significant association was observed between gender and anesthesia type (*p* > 0.05). No significant differences were found between the groups in vascular risk factors or other baseline characteristics (*p* > 0.05). Demographic characteristics are presented in Table 1, and baseline clinical, radiological, and procedural characteristics are presented in Table 2.

### 3.2. Clinical and Functional Outcomes

**Figure 2:**
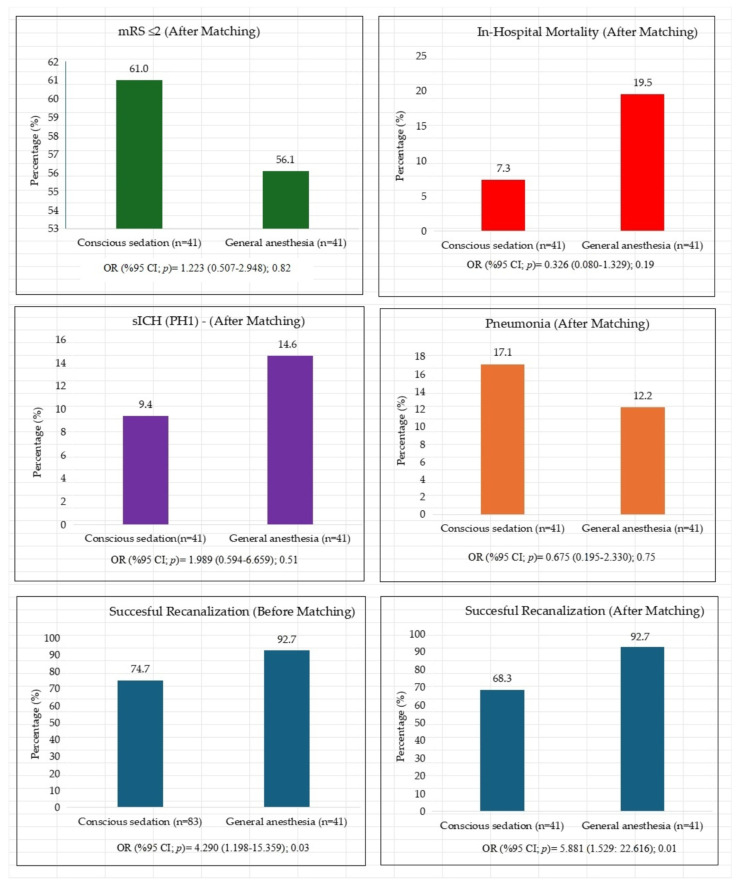
Comparison of clinical outcomes and complications between conscious sedation and general anesthesia groups after propensity score matching. Event percentages, odds ratios with 95% confidence intervals, and *p*-values are presented.

Successful recanalization (TICI ≥2b) was achieved in 100 patients. Successful recanalization was observed in 62 patients (74.7%) in the CS group and 38 patients (92.7%) in the GA group. The number of patients with successful recanalization was significantly higher in the GA group (*p* = 0.03). After Propensity Score Matching, successful recanalization was achieved in 66 patients. Successful recanalization was observed in 28 patients (68.3%) in the CS group and 38 patients (92.7%) in the GA group. The number of successfully recanalized patients remained significantly higher in the GA group (*p* = 0.01). Pneumonia developed in 28 patients overall. Pneumonia was observed in 23 patients (27.7%) in the CS group and 5 patients (12.2%) in the GA group. Although pneumonia was more frequent in the CS group, no statistically significant difference was found between the groups (*p* = 0.08). After Propensity Score Matching, pneumonia was observed in 12 patients. Pneumonia was observed in 7 patients (17.1%) in the CS group and 5 patients (12.2%) in the GA group. No significant difference was found between the groups (*p* = 0.75). Other clinical and functional outcomes were also similar between the groups (*p* > 0.05). Clinical and functional outcomes before and after matching are presented in Table 3. In addition, the comparison of clinical outcomes and complications between conscious sedation and general anesthesia groups after Propensity Score Matching is graphically presented in Figure 2.

## 4. Discussion

In this study, the effects of CS and GA on clinical outcomes were compared between patient groups undergoing MT for acute anterior circulation large vessel occlusion, following propensity score matching. Our results showed no significant differences between the CS and GA groups in mortality, functional independence, or complication rates. However, the rate of successful recanalization was significantly higher in the GA group. While anesthesia management during MT has become a standard procedure today for patients presenting with ischemic stroke, controversy persists regarding the optimal anesthetic modality.

The greater stability of procedural conditions under GA may influence the decision to proceed with or terminate the MT procedure. In the literature, GA is recommended for patients with reduced levels of consciousness, agitation, or nausea/vomiting. Furthermore, it is reported that the requirement for GA may be more frequent in patients with large infarct volumes, dominant hemisphere strokes, posterior circulation strokes, and tandem cervical-intracranial carotid lesions that may necessitate stenting [17,18].

Studies evaluating the impact of anesthetic procedures on clinical outcomes have yielded conflicting results. Some studies have reported poorer neurological outcomes in patients undergoing GA compared to those receiving CS [19,20,21]. Conversely, similar studies have found no significant differences between the GA and CS patient cohorts [22,23]. These discrepant results across studies may stem from the lack of a standardized anesthesia protocol, variations in thrombectomy techniques and devices used, differences in the experience of the anesthesiologist and interventional neurologist, and variations in hemodynamic management [7]. In our study, MT procedures were performed by a single interventional neurologist using the same thrombectomy device. The choice of anesthesia type and anesthetic agents was determined by the attending anesthesiologists. Current guidelines suggest that the choice of anesthetic technique should be tailored to the patient’s clinical status and the neurointerventionalist’s experience [24]. GA may offer advantages by ensuring patient immobilization, reducing the risk of procedural complications, and enabling a more controlled neurointerventional procedure. However, sudden drops in mean arterial pressure and the subsequent decrease in cerebral perfusion pressure raise concerns regarding GA in acute ischemic stroke patients. Additionally, mechanical ventilation-related complications are among the potential disadvantages of GA [25].

In this study, demographic data, vascular risk factors, stroke etiologies, collateral scores, ASPECTS, and NIHSS scores, MT procedure times, post-procedural complications, and the presence of hemorrhagic transformation were evaluated, all of which could influence clinical outcomes during MT and follow-up. It was observed that the choice of anesthetic technique did not result in a significant difference in peri- and post-procedural complication rates, length of hospital stay, or mRS scores during clinical follow-up. The primary finding of this observational, retrospective, single-center study is that the use of GA in patients with anterior circulation ischemic stroke was associated with higher successful reperfusion rates. This outcome is thought to be due to the optimization of procedural conditions for the interventional neurologist, including patient immobilization and airway control, despite the prolongation of the symptom-to-puncture time associated with premedication in GA patients. These optimal conditions may facilitate access to the occluded vessel, thereby increasing the likelihood of recanalization.

Every 10% increase in the rate of successful reperfusion is associated with a significant increase in the likelihood of achieving a favorable functional outcome. Increased reperfusion rates may also contribute to reductions in the rates of symptomatic intracranial hemorrhage and mortality [26]. In this study, despite the significantly higher rate of successful reperfusion, no significant difference was observed between the groups regarding functional independence during follow-up. This indicates that even when reperfusion success is high, functional outcomes do not depend solely on successful recanalization; baseline infarct volume, collateral circulation status, periprocedural hemodynamic stability, reperfusion injury, and post-stroke complications may be more decisive [26,27,28,29,30]. In particular, conditions such as hemodynamic instability, hypotension, and the resulting decrease in cerebral perfusion that can develop in patients under GA may adversely affect clinical outcomes. This may culminate in futile reperfusion, where clinical improvement is absent despite successful vessel recanalization [23,26,27,28]. A decrease in mean arterial pressure of more than 40% from baseline is an independent predictor of poor neurological outcomes [7]. In a retrospective study by Valent et al., a linear relationship was demonstrated between the duration of hypotension and adverse neurological outcomes [29]. Current literature recommends maintaining systolic blood pressure between 140–180 mmHg and mean arterial pressure at ≥70 mmHg throughout the procedure [31]. Excessive decreases in blood pressure should be avoided during MT. Therefore, maintaining hemodynamic stability during MT is of great importance [32].

In a 2023 meta-analysis of 9 randomized controlled trials and 1342 patients, a higher rate of successful reperfusion was observed in the GA group, consistent with our findings. No significant differences were detected in functional independence, procedure duration, mortality rate, or length of hospital or intensive care unit stay [11]. When pneumonia was evaluated, conflicting results have been reported in the literature. Some meta-analyses have shown a higher risk of pneumonia in patients undergoing general anesthesia [33,34]. In contrast to these studies, pneumonia was observed less frequently in the general anesthesia group in our study. However, no statistically significant difference in pneumonia rates was detected between the groups. Similar to our findings, in the matched-pair analysis conducted by Rohde et al., pneumonia was observed only in the conscious sedation group [35]. Likewise, in the large cohort analysis of the MR CLEAN study group, no marked increase in pneumonia rates was identified among patients receiving general anesthesia [36]. In both studies, no significant difference was found between the groups regarding the presence of pneumonia.

Endotracheal intubation may contribute to the prevention of aspiration and aspiration-related pulmonary complications in selected patients by providing airway protection. Nevertheless, the development of post-stroke pneumonia is influenced not only by the anesthetic technique used but also by several patient- and procedure-related factors, including stroke severity, dysphagia burden, aspiration risk, airway management strategies, and perioperative care practices [37]. In light of these considerations, it is not unexpected that the findings of our study do not show complete agreement with all previously published studies. Although more studies are needed on this topic, the current results indicate that the choice of anesthesia during MT will become an important factor influencing the clinical approach of both anesthesiologists and interventional neurologists in the future. Existing guidelines state that it is appropriate to individualize the anesthetic technique, taking into account the patient’s risk factors, the procedural technical specifications, and other clinical conditions [8].

This study has several limitations. First, the procedures at the stroke center were performed by different anesthesiologists, which may have affected standardization. The anesthesia modality was not determined in a randomized manner; instead, the type of anesthesia for each patient was determined by the attending anesthesiologist, taking into account the patient’s clinical characteristics and preprocedural condition. Although PSM analysis was applied to obtain more reliable results, the effects of some confounding factors that were unmeasured or could not be included in the model cannot be completely excluded, since it was not possible to evaluate all clinical factors. Detailed intraoperative data, such as blood pressure variability and vasopressor use, were not included in the data collection process. As these variables may affect cerebral perfusion, reperfusion success, and clinical outcomes, they should be taken into consideration when interpreting the findings. The retrospective and single-center design of the study is another important limitation. Therefore, larger prospective, randomized controlled, multicenter studies with standardized anesthesia protocols, detailed hemodynamic monitoring, and multivariable outcome modeling are needed to validate these findings. Despite these limitations, this study is important because it presents direct comparative results between GA and CS based on anesthesia choice in patients undergoing MT in Turkey.

## 5. Conclusions

In this study, the use of GA resulted in higher recanalization rates in patients with anterior circulation stroke undergoing MT. General anesthesia may be preferred because of its advantages, including ensuring patient immobility, providing airway control, and allowing the procedure to be performed under more controlled conditions. However, no differences were observed between the GA and CS groups regarding functional outcomes, mortality, or peri-procedural and post-procedural complications. These findings should be confirmed by larger multicenter, prospective, randomized controlled studies to establish whether both anesthetic approaches truly have comparable efficacy and safety profiles. Therefore, individual decision-making for selecting the anesthetic modality, considering patient clinical and laboratory characteristics alongside center experience and conditions, represents the most appropriate approach.

## Figures and Tables

**Table 1:** Comparison of demographic and baseline characteristics of stroke patients undergoing thrombectomy according to anesthesia type before and after propensity score matching.

	Before PSM				After PSM			
	Conscious Sedation	General Anesthesia	Test Statistic	*p*	Conscious Sedation	General Anesthesia	Test Statistic	*p*
Age (Years), median (min.–max.)	69 (40:86)	70 (41:89)	−0.688	0.49 ^q^	69 (45:86)	70 (41:89)	−0.033	0.97 ^q^
Gender, n (%)								
✓ Male	38 (45.8)	20 (48.8)	0.015	0.90 ^y^	19 (46.3)	20 (48.8)	0.000	1.00 ^y^
✓ Female	45 (542)	21 (51.2)			22 (53.7)	21 (51.2)		
Antiplatelet therapy, n (%)								
✓ Yes	40 (48.2)	23 (56.1)	0.406	0.52 ^y^	20 (48.8)	23 (56.1)	0.196	0.65 ^y^
✓ No	43 (51.8)	18 (43.9)			21 (51.2)	18 (43.9)		
Statin use, n (%)								
✓ No	71 (85.5)	33 (80.5)	0.212	0.64 ^y^	33 (80.5)	33 (80.5)	0.000	1.00 ^y^
✓ Yes	12 (14.5)	8 (19.5)			8 (19.5)	8 (19.5)		
HT, n (%)								
✓ No	43 (51.8)	16 (39)	1.322	0.25 ^y^	16 (39)	16 (39)	0.000	1.00 ^y^
✓ Yes	40 (48.2)	25 (61)			25 (61)	25 (61)		
DM, n (%)								
✓ No	59 (71.1)	30 (73.2)	0.001	0.97 ^y^	24 (58.5)	30 (73.2)	1.356	0.24 ^y^
✓ Yes	24 (28.9)	11 (26.8)			17 (41.5)	11 (26.8)		
HL, n (%)								
✓ No	65 (78.3)	32 (78.1)	0.000	1.00 ^y^	29 (70.7)	32 (78.1)	0.256	0.61 ^y^
✓ Yes	18 (21.7)	9 (22)			12 (29.3)	9 (22)		
CAD, n (%)								
✓ No	57 (68.7)	27 (65.9)	0.013	0.91 ^y^	28 (68.3)	27 (65.9)	0.000	1.00 ^y^
✓ Yes	26 (31.3)	14 (34.2)			13 (31.7)	14 (34.2)		
CHF, n (%)								
✓ No	75 (90.4)	34 (82.9)	---	0.25 ^x^	35 (85.4)	34 (82.9)	0.000	1.00 ^y^
✓ Yes	8 (9.6)	7 (17.1)			6 (14.6)	7 (17.1)		
AF, n (%)								
✓ No	50 (60.2)	26 (63.4)	0.021	0.88 ^y^	22 (53.7)	26 (63.4)	0.452	0.50 ^y^
✓ Yes	33 (39.8)	15 (36.6)			19 (46.3)	15 (36.6)		
Transfer from another center, n (%)								
✓ No	37 (44.6)	19 (46.3)	0.000	1.00 ^y^	19 (46.3)	19 (46.3)	0.000	1.00 ^y^
✓ Yes	46 (55.4)	22 (53.7)			22 (53.7)	22 (53.7)		
Stroke etiology, n (%)								
✓ Large artery disease	12 (14.5)	6 (14.6)	1.071	0.61 ^z^	9 (22)	6 (14.6)	2.607	0.23 ^z^
✓ Cardioembolic	68 (81.9)	35 (85.4)			30 (73.2)	35 (85.4)		
✓ Other causes	3 (3.6)	0 (0)			2 (4.9)	0 (0)		
Localization, n (%)								
✓ Right hemisphere	40 (48.2)	26 (63.4)	1.979	0.16 ^z^	22 (53.7)	26 (63.4)	0.452	0.50 ^y^
✓ Left hemisphere	43 (51.8)	15 (36.6)			19 (46.3)	15 (36.6)		

^x^ Pearson Chi-square test, ^y^ Yates correction, ^z^ Monte Carlo corrected Fisher’s Exact test, ^q^ Mann–Whitney U test, Data are presented as median (min.–max.), and n (%). AF: Atrial Fibrillation, CAD: Coronary Artery Disease, CHF: Congestive Heart Failure, DM: Diabetes Mellitus, HL: Hyperlipidemia, HT: Hypertension, min.: Minimum; max.: Maximum, PSM: Propensity Score Matching.

**Table 2:** Comparison of Clinical, Radiological, and Procedural Characteristics of Stroke Patients Undergoing Mechanical Thrombectomy According to Anesthesia Modality Before and After Propensity Score Matching.

	Before PSM				After PSM			
	Conscious Sedation	General Anesthesia	Test Statistic	*p*	Conscious Sedation	General Anesthesia	Test Statistic	*p*
Total ASPECT score, median (min.–max.)	9 (5:10)	9 (4:10)	−0.749	0.45 ^q^	9 (5:10)	9 (4:10)	−0.397	0.69 ^q^
✓ c-ASPECT, median (min.–max.)	5 (3:6)	5 (2:6)	−0.024	0.98 ^q^	5 (3:6)	5 (2:6)	−0.132	0.90 ^q^
✓ sc-ASPECT, median (min.–max.)	4 (2:4)	4 (2:4)	−1.631	0.11 ^q^	4 (2:4)	4 (2:4)	−0.992	0.32 ^q^
mTAN collateral score, n (%)								
✓ Poor	41 (49.4)	22 (53.7)	0.065	0.79 ^y^	20 (48.8)	22 (53.7)	0.049	0.82 ^y^
✓ Good	42 (50.6)	19 (46.3)			21 (51.2)	19 (46.3)		
Admission NIHSS, mean ± SD	15.81 ± 4.9	16.34 ± 3.83	−0.612	0.54 ^w^	15.54 ± 5.01	16.34 ± 3.83	−0.818	0.41 ^w^
Thrombolytic therapy, n (%)								
✓ No	46 (55.4)	26 (63.4)	0.429	0.51 ^y^	22 (53.7)	26 (63.4)	0.452	0.50 ^y^
✓ Yes	37 (44.6)	15 (36.6)			19 (46.3)	15 (36.6)		
First-pass recanalization, n (%)								
✓ No	57 (68.7)	27 (65.9)	0.013	0.91 ^y^	31 (75.6)	27 (65.9)	0.530	0.46 ^y^
✓ Yes	26 (31.3)	14 (34.2)			10 (24.4)	14 (34.2)		
Symptom-to-Puncture, median (min.–max.)	240 (120:420)	270 (120:390)	−0.847	0.39 ^q^	240 (120:360)	270 (120:390)	−0.612	0.54 ^q^
Puncture-to-Recanalization, median (min.–max.)	43 (15:155)	40 (18:85)	−1.264	0.20 ^q^	40 (15:145)	40 (18:85)	−0.028	0.97 ^q^
Symptom-to-Recanalization, median (min.–max.)	321 (150:468)	328 (145:461)	−0.035	0.97 ^q^	316 (159:445)	328 (145:461)	−0.408	0.68 ^q^

^y^ Yates correction, ^q^ Mann–Whitney U test, ^w^ Independent samples *t* test, Frequency (percentage), Data are presented as mean ± SD (standard deviation), median (min.–max.), and n (%). ASPECT: Alberta Stroke Program Early CT Score, c: cortical, min.: minute; min.: Minimum; max.: Maximum; NIHSS: National Institutes of Health Stroke Scale score, sc: subcortical, PSM: Propensity Score Matching.

**Table 3:** Comparison of clinical outcomes and complications according to anesthesia modality in stroke patients undergoing mechanical thrombectomy before and after propensity score matching.

	Before PSM				After PSM			
	Conscious Sedation	General Anesthesia	Test Statistic	*p*	Conscious Sedation	General Anesthesia	Test Statistic	*p*
mRS ≤ 2, n (%)	44 (53)	23 (56.1)	0.018	0.89 ^y^	25 (61)	23 (56.1)	0.050	0.82 ^y^
mRS > 2, n (%)	39 (47)	18 (43.9)			16 (39)	18 (43.9)		
In-hospital mortality, n (%)								
No	64 (77.1)	33 (80.5)	0.039	0.84 ^y^	38 (92.7)	33 (80.5)	1.680	0.19 ^y^
Yes	19 (22.9)	8 (19.5)			3 (7.3)	8 (19.5)		
Length of hospital stay (days), median (min.–max.)	18 (3:103)	18 (4:92)	−0.284	0.77^q^	22 (6:90)	18 (4:92)	−0.849	0.39 ^q^
Pneumonia, n (%)								
No	60 (72.3)	36 (87.8)	2.944	0.08 ^y^	34 (82.9)	36 (87.8)	0.098	0.75 ^y^
Yes	23 (27.7)	5 (12.2)			7 (17.1)	5 (12.2)		
Recanalization grade, n (%)								
TICI 2a and below	21 (25.3)	3 (7.3)	4.593	0.03 ^y^	13 (31.7)	3 (7.3)	6.290	0.01 ^y^
TICI 2b, 2c, or 3	62 (74.7)	38 (92.7)			28 (68.3)	38 (92.7)		
Embolization to a different vessel, n (%)								
No	77 (92.8)	39 (95.1)	---	1.00 ^z^	38 (92.7)	39 (95.1)	---	1.00 ^z^
Yes	6 (7.2)	2 (4.9)			3 (7.3)	2 (4.9)		
Perforation, n (%)								
No	82 (98.8)	41 (100)	---	1.00 ^z^	---	---	---	---
Yes	1 (1.2)	0			---	---		
Dissection, n (%)								
No	82 (98.8)	41 (100)	---	1.00 ^z^	---	---	---	---
Yes	1 (1.2)	0 (0)			---	---		
sİCH, n (%)								
None	72 (86.8)	34 (82.9)	2.018	0.51 ^t^	37 (90.2)	34 (82.9)	1.475	0.51 ^z^
PH1	11 (13.3)	6 (14.6)			4 (9.8)	6 (14.6)		
PH2	0 (0)	1 (2.4)			0 (0)	1 (2.4)		
NIHSS at 24 h, median (min.–max.)	12 (2: 25) /	12 (5: 23)	−0.855	0.39 ^q^	10 (2:25)	12 (5:23)	−0.946	0.34 ^q^
Decompressive surgery, n (%)								
No	75 (90.4)	35 (85.4)	---	0.54 ^z^	40 (97.6)	35 (85.4)	---	0.10 ^z^
Yes	8 (9.6)	6 (14.6)			1 (2.4)	6 (14.6)		

^y^ Yates correction, ^z^ Fisher’s Exact test, ^t^ Monte Carlo-corrected Fisher’s Exact test, ^q^ Mann–Whitney U test, Frequency (percentage). Data are presented as median (min.–max.), and n (%). mRS: Modified Rankin Scale, NIHSS: National Institutes of Health Stroke Scale score, sICH: Symptomatic Intracerebral Hemorrhage, PH: Parenchymal Hematoma, TICI: Thrombolysis in Cerebral Infarction, PSM: Propensity Score Matching.

## Data Availability

Data are available from the corresponding author upon request.

## Supplementary Materials

The following supporting information can be downloaded at: https://www.mdpi.com/article/10.3390/jcm15134916/s1, Supplementary Table S1: Covariate Balance Before and After Propensity Score Matching.

## References

[1] Chugh C. (2019). Acute Ischemic Stroke: Management Approach. Indian J. Crit. Care Med..

[2] Krishnamurthi R.V., Feigin V.L., Forouzanfar M.H., Mensah G.A., Connor M., Bennett D.A., Moran A.E., Sacco R.L., Anderson L.M., Truelsen T. (2013). Global and Regional Burden of First-Ever Ischaemic and Haemorrhagic Stroke during 1990–2010: Findings from the Global Burden of Disease Study 2010. Lancet Glob. Health.

[3] Elfil M., Ghaith H.S., Elsayed H., Aladawi M., Elmashad A., Patel N., Medicherla C., El-Ghanem M., Amuluru K., Al-Mufti F. (2024). Intravenous Thrombolysis Plus Mechanical Thrombectomy versus Mechanical Thrombectomy Alone for Acute Ischemic Stroke: A Systematic Review and Updated Meta-Analysis of Clinical Trials. Interv. Neuroradiol..

[4] Khoury N.N., Darsaut T.E., Ghostine J., Deschaintre Y., Daneault N., Durocher A., Lanthier S., Pope A.Y., Odier C., Lebrun L.-H. (2017). Endovascular Thrombectomy and Medical Therapy versus Medical Therapy Alone in Acute Stroke: A Randomized Care Trial. J. Neuroradiol..

[5] Matsukawa H., Crosa R., Cunningham C., Maier I., Al Kasab S., Jabbour P., Kim J.-T., Wolfe S.Q., Rai A., Starke R.M. (2024). Earlier Endovascular Thrombectomy and Mortality in Patients with Anterior Circulation Large Vessel Occlusion: A Propensity-Matched Analysis of the Stroke Thrombectomy and Aneurysm Registry. World Neurosurg..

[6] Schönenberger S., Löwhagen Hendén P., Simonsen C.Z., Uhlmann L., Klose C., Pfaff J.A., Yoo A.J., Sørensen L.H., Ringleb P.A., Wick W. (2019). Association of General Anesthesia vs Procedural Sedation with Functional Outcome Among Patients With Acute Ischemic Stroke Undergoing Thrombectomy: A Systematic Review and Meta-analysis. JAMA.

[7] Dinsmore J.E., Tan A. (2022). Anaesthesia for Mechanical Thrombectomy. Anaesthesia.

[8] Hassan A.E., Chaudhry S.A., Zacharatos H., Khatri R., Akbar U., Suri M.F.K., Qureshi A.I. (2012). Increased Rate of Aspiration Pneumonia and Poor Discharge Outcome Among Acute Ischemic Stroke Patients Following Intubation for Endovascular Treatment. Neurocrit. Care.

[9] Pop R., Severac F., Happi Ngankou E., Harsan O., Martin I., Mihoc D., Manisor M., Simu M., Chibbaro S., Wolff V. (2021). Local Anesthesia versus General Anesthesia during Endovascular Therapy for Acute Stroke: A Propensity Score Analysis. J. Neurointerv. Surg..

[10] Molina C.A., Selim M.H. (2010). General or Local Anesthesia During Endovascular Procedures: Sailing Quiet in the Darkness or Fast Under a Daylight Storm. Stroke.

[11] Geraldini F., Diana P., Fregolent D., De Cassai A., Boscolo A., Pettenuzzo T., Sella N., Lupelli I., Navalesi P., Munari M. (2023). General Anesthesia or Conscious Sedation for Thrombectomy in Stroke Patients: An Updated Systematic Review and Meta-analysis. Can. J. Anaesth..

[12] Campbell D., Butler E., Campbell R.B., Ho J., Barber P.A. (2023). General Anesthesia Compared with Non-GA in Endovascular Thrombectomy for Ischemic Stroke: A Systematic Review and Meta-analysis of Randomized Controlled Trials. Neurology.

[13] Hacke W., Kaste M., Bluhmki E., Brozman M., Dávalos A., Guidetti D., Larrue V., Lees K.R., Medeghri Z., Machnig T. (2008). Thrombolysis with Alteplase 3 to 4.5 Hours after Acute Ischemic Stroke. N. Engl. J. Med..

[14] Adams H.P., Bendixen B.H., Kappelle L.J., Biller J., Love B.B., Gordon D.L., Marsh E.E. (1993). Classification of Subtype of Acute Ischemic Stroke: Definitions for Use in a Multicenter Clinical Trial. TOAST: Trial of Org 10172 in Acute Stroke Treatment. Stroke.

[15] Higashida R.T., Furlan A.J., Roberts H., Tomsick T., Connors B., Barr J., Dillon W., Warach S., Broderick J., Tilley B. (2003). Trial Design and Reporting Standards for Intra-Arterial Cerebral Thrombolysis for Acute Ischemic Stroke. Stroke.

[16] Öner Eyüboğlu F., Bacakoglu F., Akalin H., Cakir Edis E., Cicek C., Ergan B., Eryuksel E., Gencer S., Gulay Z., Gur D. (2018). Consensus Report for the Diagnosis and Management of Hospital-Acquired Pneumonia in Adults. Turk. Thorac. J..

[17] Steinberg J.A., Somal J., Brandel M.G., Kang K.M., Wali A.R., Rennert R.C., Santiago-Dieppa D.R., Olson S.E., Pannell J.S., Khalessi A.A. (2021). Site of Occlusion May Influence Decision to Perform Thrombectomy Under General Anesthesia or Conscious Sedation. J. Neurosurg. Anesthesiol..

[18] Hindman B.J., Dexter F. (2019). Anesthetic Management of Emergency Endovascular Thrombectomy for Acute Ischemic Stroke, Part 2: Integrating and Applying Observational Reports and Randomized Clinical Trials. Anesth. Analg..

[19] Just C., Rizek P., Tryphonopoulos P., Pelz D., Arango M. (2016). Outcomes of General Anesthesia and Conscious Sedation in Endovascular Treatment for Stroke. Can. J. Neurol. Sci..

[20] Berkhemer O.A., van den Berg L.A., Fransen P.S.S., Beumer D., Yoo A.J., Lingsma H.F., Schonewille W.J., Berg R.V.D., Wermer M.J.H., Boiten J. (2016). MR CLEAN Investigators. The Effect of Anesthetic Management During Intra-Arterial Therapy for Acute Stroke in MR CLEAN. Neurology.

[21] Goldhoorn R.J.B., Bernsen M.L.E., Hofmeijer J.M., Martens J.M., Lingsma H.F., Dippel D.W., van der Lugt A., Buhre W.F., Roos Y.B., Majoie C.B. (2020). Anesthetic Management during Endovascular Treatment of Acute Ischemic Stroke in the MR CLEAN Registry. Neurology.

[22] Schönenberger S., Uhlmann L., Hacke W., Schieber S., Mundiyanapurath S., Purrucker J.C., Nagel S., Klose C., Pfaff J., Bendszus M. (2016). Effect of Conscious Sedation vs General Anesthesia on Early Neurological Improvement Among Patients With Ischemic Stroke Undergoing Endovascular Thrombectomy: A Randomized Clinical Trial. JAMA.

[23] Löwhagen Hendén P., Rentzos A., Karlsson J.E., Rosengren L., Leiram B., Sundeman H., Dunker D., Schnabel K., Wikholm G., Hellström M. (2017). General Anesthesia Versus Conscious Sedation for Endovascular Treatment of Acute Ischemic Stroke: The AnStroke Trial (Anesthesia During Stroke). Stroke.

[24] Powers W.J., Rabinstein A.A., Ackerson T., Adeoye O.M., Bambakidis N.C., Becker K., Biller J., Brown M., Demaerschalk B.M., Hoh B. (2019). Guidelines for the Early Management of Patients With Acute Ischemic Stroke: 2019 Update to the 2018 Guidelines for the Early Management of Acute Ischemic Stroke: A Guideline for Healthcare Professionals From the American Heart Association/American Stroke Association. Stroke.

[25] Turkish Neurological Society Stroke Study Group (2020). Acute Ischemic Stroke Diagnosis and Treatment Guideline 2020.

[26] Manning N.W., Warne C.D., Meyers P.M. (2018). Reperfusion and Clinical Outcomes in Acute Ischemic Stroke: Systematic Review and Meta-Analysis of the Stent-Retriever-Based, Early Window Endovascular Stroke Trials. Front. Neurol..

[27] Jagani M., Brinjikji W., Rabinstein A.A., Pasternak J.J., Kallmes D.F. (2016). Hemodynamics during Anesthesia for Intra-Arterial Therapy of Acute Ischemic Stroke. J. Neurointerv. Surg..

[28] Lee S.H., Kim B.J., Han M.K., Park T.H., Lee K.B., Lee B.C., Yu K.-H., Oh M.S., Cha J.K., Kim D.-H. (2019). Futile Reperfusion and Predicted Therapeutic Benefits after Successful Endovascular Treatment According to Initial Stroke Severity. BMC Neurol..

[29] Valent A., Sajadhoussen A., Maier B., Lapergue B., Labeyrie M.-A., Reiner P., Consoli A., Fischler M., Gayat E., Leguen M. (2020). A 10% Blood Pressure Drop from Baseline During Mechanical Thrombectomy for Stroke Is Strongly Associated with Worse Neurological Outcomes. J. Neurointerv. Surg..

[30] Bang O.Y., Saver J.L., Kim S.J., Kim G.M., Chung C.S., Ovbiagele B., Lee K.H., Liebeskind D.S. (2011). Collateral Flow Predicts Response to Endovascular Therapy for Acute Ischemic Stroke. Stroke.

[31] Talke P.O., Sharma D., Heyer E.J., Bergese S.D., Blackham K.A., Stevens R.D. (2014). Society for Neuroscience in Anesthesiology and Critical Care Expert Consensus Statement: Anesthetic Management of Endovascular Treatment for Acute Ischemic Stroke: Endorsed by the Society of NeuroInterventional Surgery and the Neurocritical Care Society. J. Neurosurg. Anesthesiol..

[32] Wiącek M., Tomaszewska-Lampart I., Dziedzic M., Kaczorowska A., Bartosik-Psujek H. (2024). Association between Transient-Continuous Hypotension during Mechanical Thrombectomy for Acute Ischemic Stroke and Final Infarct Volume in Patients with Proximal Anterior Circulation Large Vessel Occlusion. J. Clin. Med..

[33] Peng Z., Luo W., Yan Z., Zhang H. (2023). The Effect of General Anesthesia and Conscious Sedation in Endovascular Thrombectomy for Acute Ischemic Stroke: An Updated Meta-Analysis of Randomized Controlled Trials and Trial Sequential Analysis. Front. Neurol..

[34] Zhao J., Tan X., Wu X., Li J., Wang S., Qu R., Qu R., Chu T., Chen Z., Liu J. (2023). The Efficacy and Safety of General Anesthesia vs. Conscious Sedation for Endovascular Treatment in Patients with Acute Ischemic Stroke: A Systematic Review and Meta-Analysis. Front. Neurol..

[35] Rohde S., Schwarz S., Alexandrou M., Reimann G., Ellerkmann R.K., Politi M. (2019). Effect of General Anaesthesia versus Conscious Sedation on Clinical and Procedural Outcome in Patients Undergoing Endovascular Stroke Treatment: A Matched-Pair Analysis. Cerebrovasc. Dis..

[36] van den Berg L.A., Koelman D.L.H., Berkhemer O.A., Rozeman A.D., Fransen P.S.S., Beumer D., Dippel D.W., van der Lugt A., van Oostenbrugge R.J., van Zwam W.H. (2015). Type of Anesthesia and Differences in Clinical Outcome after Intra-Arterial Treatment for Ischemic Stroke. Stroke.

[37] Talke P.O., Sharma D., Heyer E.J., Bergese S.D., Blackham K.A., Stevens R.D. (2014). Republished: Society for Neuroscience in Anesthesiology and Critical Care Expert Consensus Statement: Anesthetic Management of Endovascular Treatment for Acute Ischemic Stroke. Stroke.

