# Supplementary material for: Impact of Anesthesia Modality on Clinical Outcomes in Anterior Circulation Stroke Patients Undergoing Mechanical Thrombectomy: A Retrospective Propensity Score-Matched Analysis
Source: J Clin Med. 2026 Jun 24;15(13):4916. doi: 10.3390/jcm15134916 (PMC13361245; doi:10.3390/jcm15134916)
Supplement: Supplementary file 1 [file jcm-15-04916-s001.zip › jcm-4354207-supplementary.pdf]

Table S1. Covariate Balance Before and After Propensity Score Matching

Variable	Before PSM Conscious Sedation (n=83)	Before PSM General Anesthesia (n=41)	Standardized Mean Difference (Before)	After PSM Conscious Sedation (n=41)	After PSM General Anesthesia (n=41)	Standardized Mean Difference (After)
Sex (Male)	38 (45,8)	20 (48,8)	0,060	19 (46,3)	20 (48,8)	0,049
Antiplatelet therapy (Yes)	40 (48,2)	23 (56,1)	0,159	20 (48,8)	23 (56,1)	0,147
Statin use (Yes)	12 (14,5)	8 (19,5)	0,135	8 (19,5)	8 (19,5)	0,000
Hypertension (Yes)	40 (48,2)	25 (61,0)	0,259	25 (61,0)	25 (61,0)	0,000
Diabetes mellitus (Yes)	24 (28,9)	11 (26,8)	0,047	17 (41,5)	11 (26,8)	0,312
Hyperlipidemia (Yes))	18 (21,7)	9 (22,0)	0,006	12 (29,3)	9 (22,0)	0,168
Coronary artery disease (Yes)	26 (31,3)	14 (34,1)	0,060	13 (31,7)	14 (34,1)	0,052
Heart failure (Yes))	8 (9,6)	7 (17,1)	0,220	6 (14,6)	7 (17,1)	0,067
Atrial fibrillation (Yes)	33 (39,8)	15 (36,6)	0,065	19 (46,3)	15 (36,6)	0,199
Interhospital transfer (Yes)	46 (55,4)	22 (53,7)	0,035	22 (53,7)	22 (53,7)	0,000
Etiology (Large artery atherosclerosis)	12 (14,5)	6 (14,6)	0,005	9 (22,0)	6 (14,6)	0,190

Etiology (Cardioembolism)	68 (81,9)	35 (85,4)	0,093	30 (73,2)	35 (85,4)	0,304
Etiol)ogy (Other determined etiology	3 (3,6)	0 (0,0)	0,274	2 (4,9)	0 (0,0)	0,320
Occlusion side (Right hemisphere)	40 (48,2)	26 (63,4)	0,310	22 (53,7)	26 (63,4)	0,199
Occlusion side (Left hemisphere)	43 (51,8)	15 (36,6)	0,310	19 (46,3)	15 (36,6)	0,199
m_TAN (Poor)	41 (49,4)	22 (53,7)	0,085	20 (48,8)	22 (53,7)	0,098
Intravenous thrombolysis (Yes)	37 (44,6)	15 (36,6)	0,163	19 (46,3)	15 (36,6)	0,199
First-pass effect (Yes)	26 (31,3)	14 (34,1)	0,060	10 (24,4)	14 (34,1)	0,216
Age	66,99±12,43 / n=83	69,20±10,63 / n=41	0,186	69,17±10,16 / n=41	69,20±10,63 / n=41	0,002
ASPECT	8,81±1,36 / n=83	8,54±1,61 / n=41	0,187	8,61±1,53 / n=41	8,48±1,60 / n=41	0,083
c_ASPECT	5,17±0,95 / n=83	5,12±1,10 / n=41	0,050	5,05±1,05 / n=41	5,08±1,07 / n=41	0,028
sc_ASPET	3,63±0,68 / n=83	3,41±0,71 / n=41	0,319	3,54±0,74 / n=41	3,40±0,71 / n=41	0,193
Admission NIHSS	15,81±4,90 / n=83	16,34±3,82 / n=41	0,117	15,54±5,01 / n=41	16,34±3,82 / n=41	0,181

Symptom-to-puncture time (min)	251,14±71,99 / n=83	260,49±81,66 / n=41	0,124	252,07±70,29 / n=41	260,49±81,66 / n=41	0,110
Puncture-to-recanalization time (min)	56,53±34,40 / n=83	44,17±17,94 / n=41	0,412	46,95±26,83 / n=41	44,17±17,94 / n=41	0,122
Symptom-to-recanalization time (min)	307,64±79,53 / n=83	304,17±86,87 / n=41	0,042	299,07±74,63 / n=41	304,17±86,87 / n=41	0,063
Length of hospital stay (days)	25,78±24,88 / n=83	23,37±18,90 / n=41	0,105	26,41±19,86 / n=41	23,37±18,90 / n=41	0,157
24-hour NIHSS score	12,22±6,02 / n=83	13,02±5,66 / n=41	0,137	11,83±5,95 / n=41	13,02±5,66 / n=41	0,206

Frekans (yüzde); ortalamam±s. sapma / n;

ASPECT: Alberta Stroke Program Early CT

c: Cort□cal ASPECTS

NIHSS: National Institutes of Health Stroke Scale score

Sc: Subcortical ASPECTS
